# Supplementary figures and images for: Genome-based analysis of infrequent Salmonella serotypes through the Thai pork production chain
Source: Front Microbiol. 2022 Aug 25;13:968695. doi: 10.3389/fmicb.2022.968695 (PMC9453559; doi:10.3389/fmicb.2022.968695)

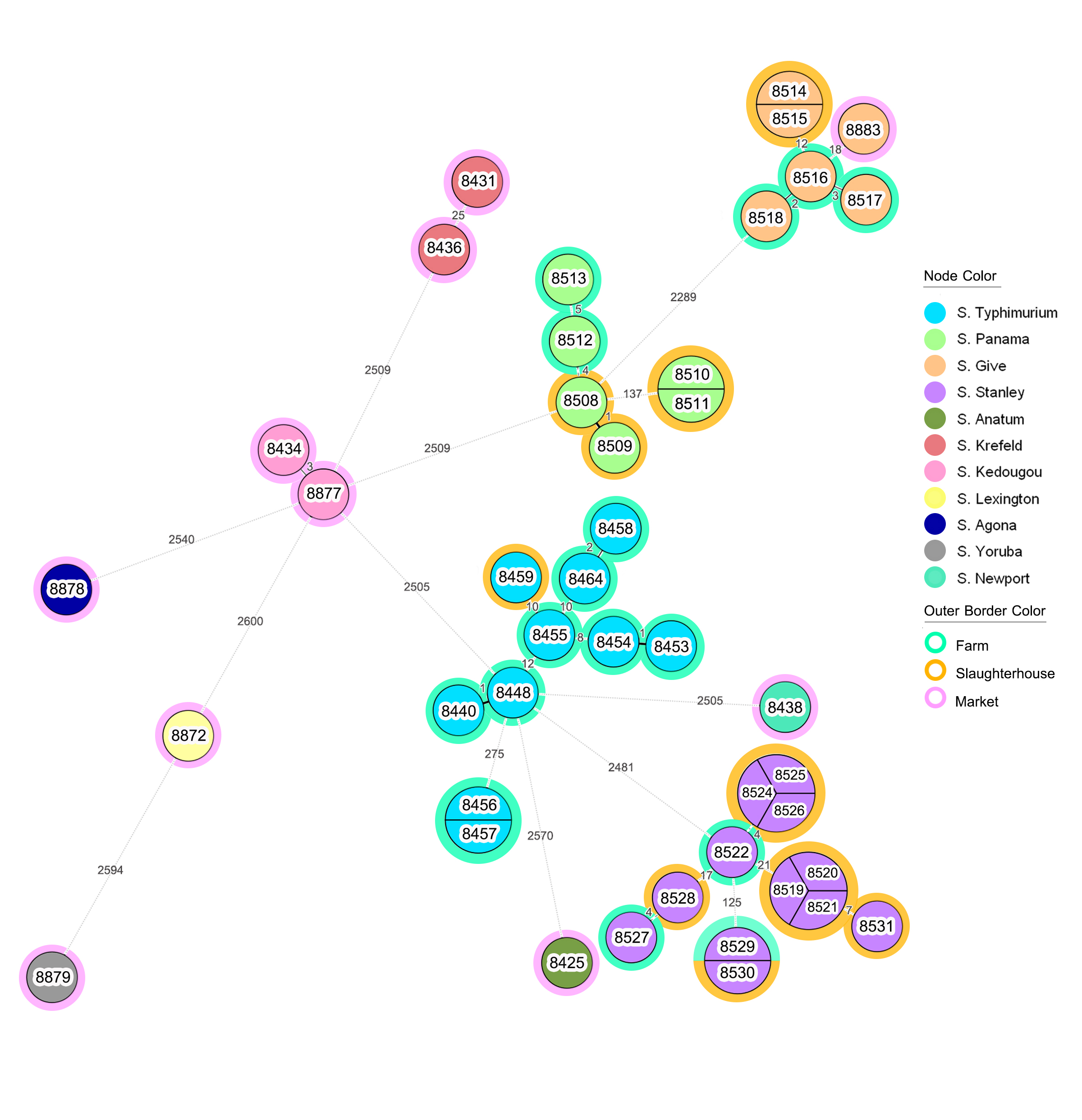

Supplement: Supplementary Figure 1 — The minimum spanning tree (MST) analysis of Salmonella isolates recovered from the pork production chain. Each isolate was grouped according to the EnteroBase Salmonella database’s cgMLST scheme, which considers a total of 3,002 loci (Pearce et al., 2020). The number on the branch represent the number of loci different between each isolate. Node color represents each serotype of Salmonella isolates. Outer border color coding: green color, yellow color and pink color represent the Salmonella isolates recovered from farm, slaughterhouse and retail market, respectively. [file Image_1.JPEG]

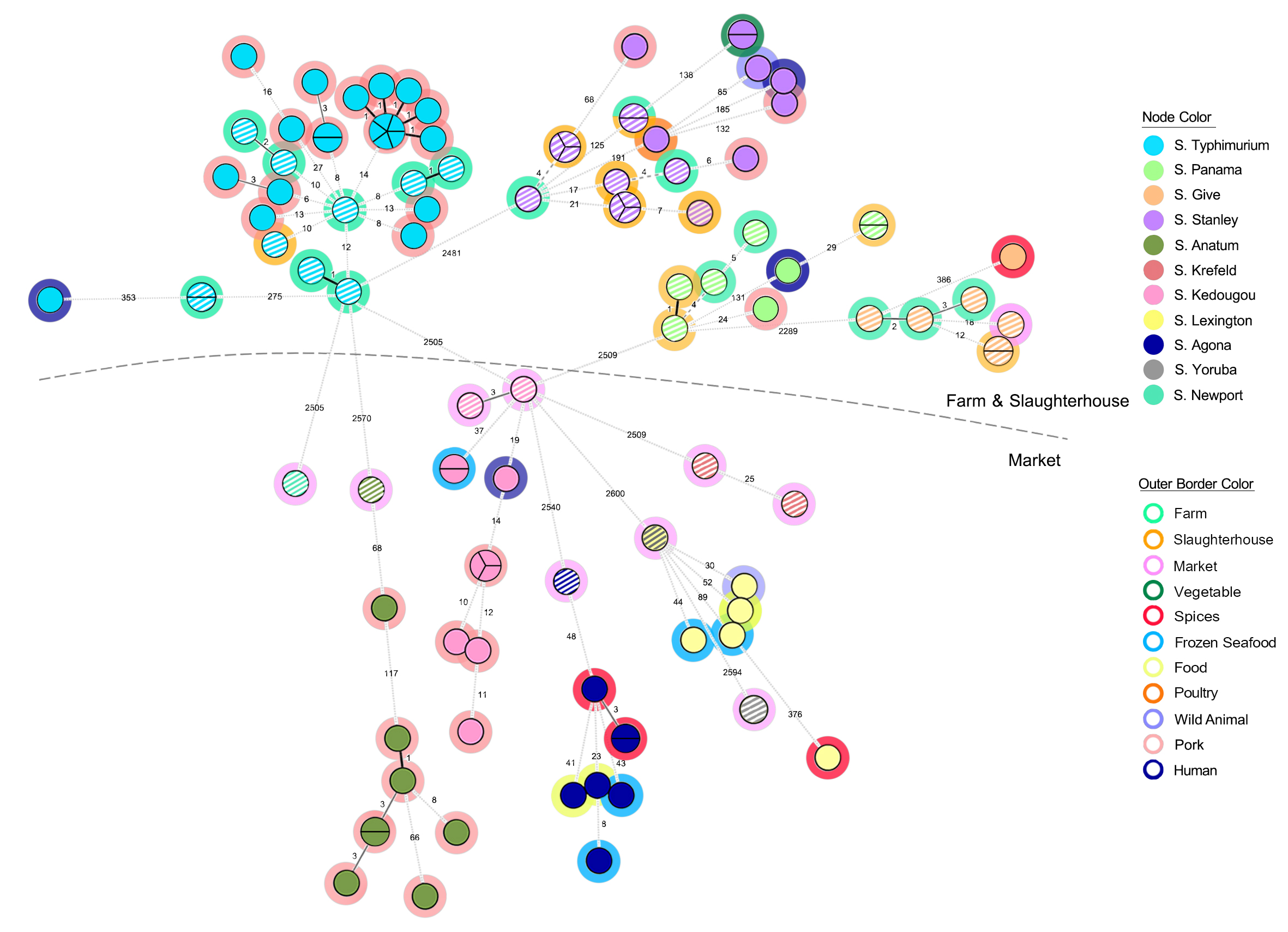

Supplement: Supplementary Figure 2 — The minimum spanning tree (MST) analysis of 43 Salmonella isolates recovered from pork production chain (striped nodes) and additional 61 Salmonella isolates circulating in Thailand. Each isolate was grouped according to the loci different of EnteroBase Salmonella database’s cgMLST scheme, which considers a total of 3,002 loci (Pearce et al., 2020). The number on the branch represent the number of loci different between each isolate. Node color coding were representing the serotypes of the Salmonella isolates. Outer border color represents each source of Salmonella isolates. [file Image_2.jpg]

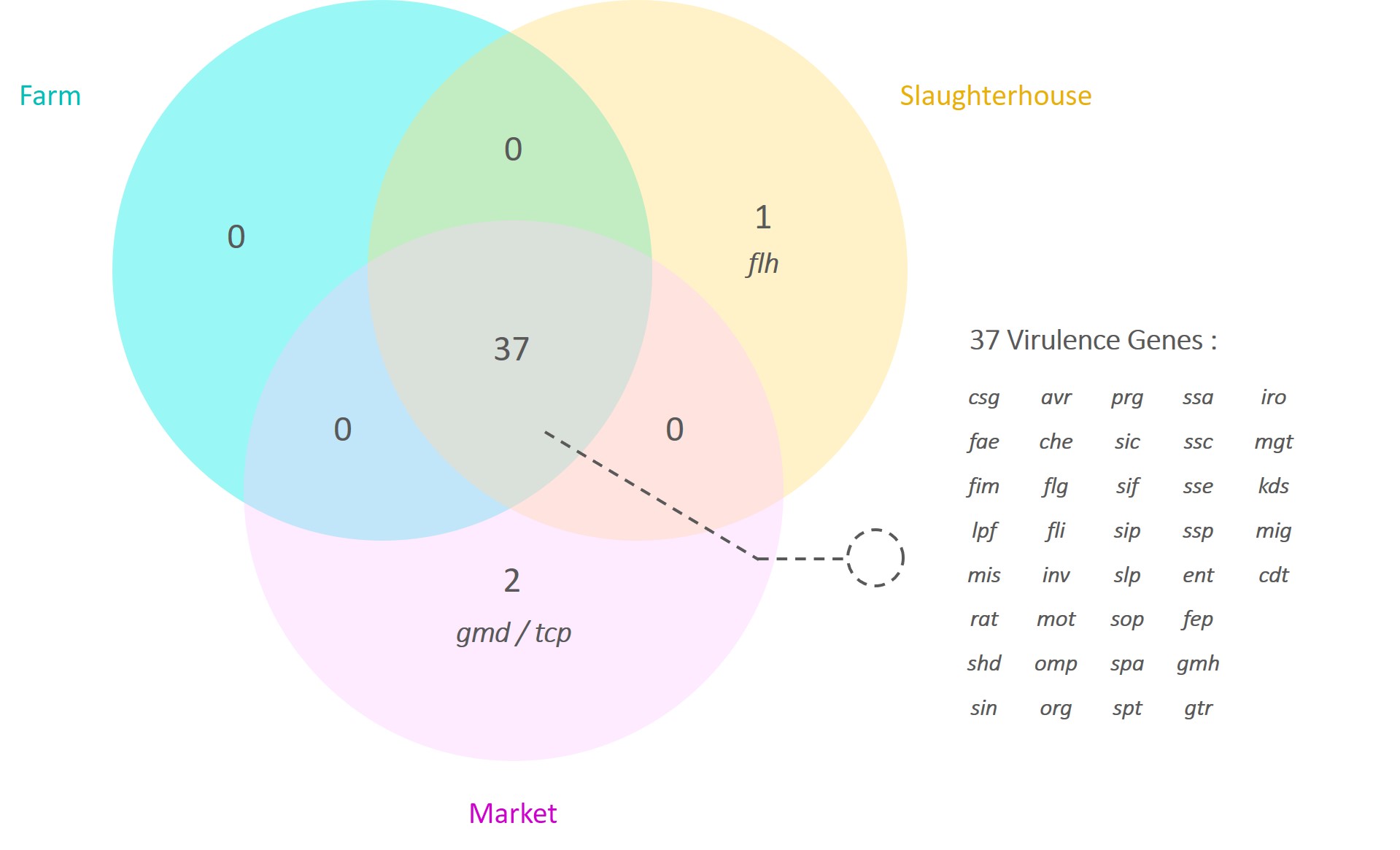

Supplement: Supplementary Figure 3 — The Venn diagram of intersection analysis of virulence genes among different steps of pork production chain. The Venn diagram represent the number of unique and shared virulence genes in 43 Salmonella isolates recovered from farms, slaughterhouses and retail markets. [file Image_3.JPEG]

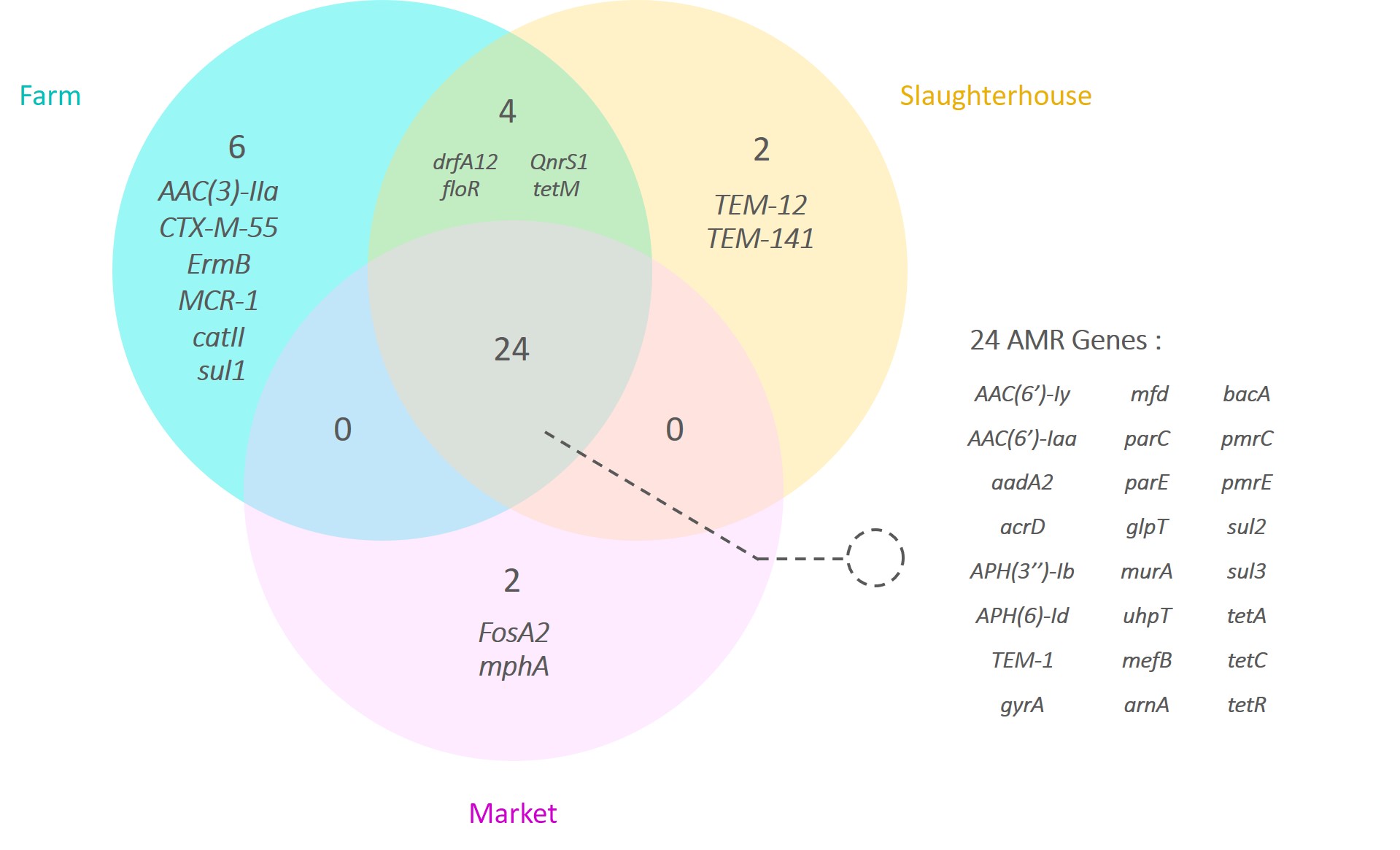

Supplement: Supplementary Figure 4 — The Venn diagram of intersection analysis of antimicrobial resistance genes among different steps of the pork production chain. The Venn diagram represents the number of unique and shared antimicrobial resistance genes in 43 Salmonella isolates recovered from farms, slaughterhouses, and retail markets. [file Image_4.JPEG]
